# A review on the ecological niche and functional properties of enterococci in health promotion

**DOI:** 10.1016/j.crfs.2025.101211

**Published:** 2025-09-25

**Authors:** Yao He, Zhigao Liu, Yina Huang, Liang Qiu, Xueying Tao, Hua Wei

**Affiliations:** a State Key Laboratory of Food Science and Resources, Nanchang University, Nanchang, Jiangxi, 330047, PR China; b Jiangxi-OAI Joint Research Institute, Nanchang University, Nanchang, Jiangxi, 330047, PR China; c International Institute of Food Innovation Co., Ltd., Nanchang University, Nanchang, Jiangxi, 330052, PR China; d College of Biological and Food Engineering, Anhui Polytechnic University, Wuhu, Anhui, 241000, PR China; e Centre for Translational Medicine, Jiangxi University of Traditional Chinese Medicine, Nanchang, Jiangxi, 330006, PR China

**Keywords:** Enterococci, Biological niches, Functional *metabolites*, Starter-cultures, Promotion effects

## Abstract

Enterococci are ubiquitous lactic acid bacteria (LAB) that mainly inhabit the gastrointestinal tracts of humans and animals and are also widely present in foods and the environment due to their robust survival capacity. While many studies have focused on their pathogenic potential, increasing attention is being given to their probiotic properties. In this review, we summarized the ecological niches of enterococci in the genus, habitats, and food systems, and comprehensively discuss their functional metabolites, including organic acids, enterocins, exopolysaccharides, and surface layer proteins, as well as their roles in fermentation and health promotion, such as inhibiting *Listeria monocytogenes*, alleviating metabolic disorders, hypercholesterolemia, and cancer development. Importantly, unlike previous reviews that emphasized pathogenesis with only a brief mention of probiotic traits, this review provides an integrated and mechanism-oriented perspective on enterococci as probiotics, and highlights future research directions, including their interactions with food matrices and host gastrointestinal systems, as well as the application of artificial intelligence and machine learning for the rigorous screening of safe enterococcal probiotics.

## Highlights

• Functional metabolites from enterococci include organic acids, enterocins, EPS, and S-layer proteins.
• Enterococci as starter-cultures enhance flavor and show antimicrobial activity against pathogens, notably *L*. *monocytogenes.*
• Health benefits of enterococci involve the alleviation of cancer, metabolic syndrome, and hypercholesterolemia, *etc.*

## Graphical abstract

**Figure:**
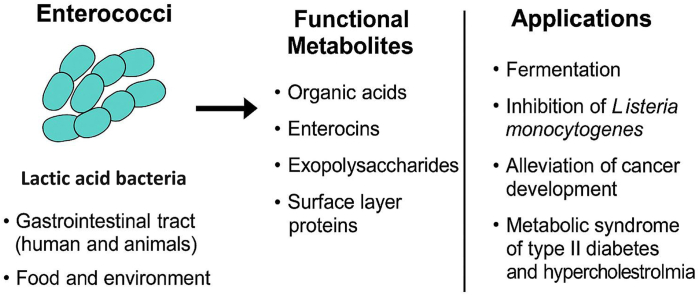

## 1 Introduction

Probiotics are defined as live microorganisms that, when administered in adequate amounts, confer health benefits to the host, such as enhancing the immune system, reducing metabolic disorders, and improving feed digestibility. Their screening and characterization mainly focus on properties such as survival during oral-gastrointestinal transit, production of antimicrobial substances, antibiotic susceptibility, adhesion to the human intestinal mucosa, and desirable immunomodulatory activity. These evaluations are largely based on standards established by the Food and Agriculture Organization (FAO) and the World Health Organization (WHO) (Ganguly et al., 2011) (Sanders et al., 2010). Therein, plenty of lactic acid bacteria (LAB) from the human gastrointestinal tract were recommended and generally recognized as safe (GRAS), among which *Bifidobacteria* and *lactobacilli* were more commonly used. LAB strains isolated from animals, fermented or non-fermented foods, may also be potential probiotic candidates, for instance, *Enterococcus* (Pogány Simonová et al., 2020), *Carnobacterium* (Evangelista et al., 2023) and *Akkermansia muciniphila* (Cani et al., 2022) presented their advantages of technical processing and health-promoting properties.

Enterococci constitute a large proportion of indigenous bacteria associated with the mammalian gastrointestinal tract and fermented foods, which are predominantly composed of *Enterococcus faecalis*, followed by *Enterococcus faecium*, *Enterococcus durans*, and *Enterococcus hirae* (Franz et al., 2003). As one of the gut commensal microbiotas, enterococci intrinsically involved in the incredibly complex web of metabolic relations with other gut inhabitants, eukaryotic host, and gastrointestinal immune system, leading to be a potential candidate for treating several diseases such as irritable bowel syndrome, diarrhea or antibiotic associated diarrhea, or for health improvement such as lowering cholesterol levels or immune regulation (Franz et al., 2011). Besides, by yielding natural lactate and ribosomal-synthesized proteinaceous substances e.g., bacteriocins, which kill many food spoilage microorganisms and some food-borne pathogens, enterococci were suitable for application as food additives *in situ* or *ex situ* to prolong the shelf-life in the food industry (Foulquié Moreno et al., 2006). Apart from using as a starter culture, fermentation with milk or some food and drug homology (lotus leaf, ginseng, *etc.*) by enterococci also contributed to varying benefits, e.g., ameliorating hypertension, obesity and type II diabetes, *etc.,* mainly *via* producing angiotensin converting enzyme inhibitor (ACEI), flavonoids and polyphenols after fermentation (He et al., 2022) (Xie et al., 2020). Conversely, nosocomial infection of enterococci was well documented for their multiple-antibiotic resistance, especially vancomycin-resistant enterococci, resulting in a dispute on the utilization of enterococci as probiotic supplements (Graham et al., 2020). Namely, enterococci have a paradoxical position, as they can be applied in the food industry while also posing some risk to human health. To date, to our limited knowledge, certain *Enterococcus* strains isolated from healthy individuals or traditional fermented foods, which lack putative virulence determinants, have been recognized for their probiotic potential. Notably, *E. faecium* strain K77D, originally isolated from the gut microbiota of long-lived individuals in Abkhazia and exhibiting good probiotic potential, has been approved by the UK Advisory Committee on Novel Foods and Processes (ACNFP) for use as a starter culture in fermented dairy products, and has also been accepted for use during cheese ripening in Denmark (Hanchi et al., 2018). It serves as the core probiotic strain in the fermented milk product Gaio® with two other *Streptococcus thermophilus* strains to support fermentation and improve texture and flavor, combining technological performance with potential health benefits in serum cholesterol, diarrhea, and mutagens (Bertolami et al., 2003).

Unlike existing reviews that primarily focus on the pathogenic aspects of enterococci and provide only a short overview of their probiotic features, this review emphasizes their ecological niches, functional metabolites, and potential health-promoting mechanisms. Specifically, we provide a systematic summary of organic acids, enterocins, exopolysaccharides, and surface layer proteins produced by enterococci, and evaluate their applications in food fermentation and human health, including inhibition of *Listeria monocytogenes*, mitigation of metabolic syndrome and type II diabetes, reduction of hypercholesterolemia, and modulation of cancer development. In addition, we point to future research directions, including the need to elucidate their interactions with food matrices and host gastrointestinal systems, and the use of artificial intelligence and machine learning to accelerate the rigorous screening and functional evaluation of safe enterococcal strains. By integrating ecological, functional, and mechanistic insights, this review offers a novel perspective on the potential of enterococci as probiotics for food applications and health promotion.

## 2 The genus enterococci

**Fig. 1:**
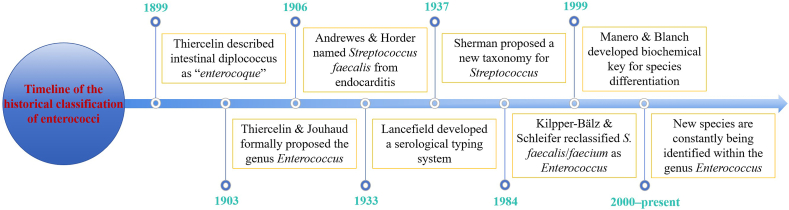
Timeline of the historical classification of enterococci. Major milestones from its first description in 1899 to the genomic era are shown.

The timeline of enterococci taxonomy is shown in Fig. 1. Thiercelin first described the Gram-positive diplococcus associated with the gastrointestinal tract of humans and animals with the term “*enterocoque*” in 1899 (Thiercelin and Jouhaud, 1899); the genus Enterococcus was proposed by Thiercelin and Jouhaud in 1903, which comes from the Greek words “entero” (“έ*ντερο*”) meaning “intestine” and “coccus” (“*κ*ό*κκος*”) meaning “spherical particle,” perfectly describing their origin and morphology together (Thiercelin and Jouhaud, 1903). However, in 1906, Anby drewes and Horder classified potentially pathogenic bacteria from a patient with endocarditis as Streptococcus faecalis, in which “faecalis” was suggested because of the close resemblance with strains isolated from intestinal origin (Andrewes and Horder, 1906). Classical taxonomy of enterococci still faces many challenges and remains rather vague because of a lack of clear phenotypical criteria that can explicitly distinguish enterococci from other Gram-positive, catalase-negative, coccus-shaped bacterial genera. In 1933, Lancefield developed a serological typing system for enterococci whereby those of “fecal origin” possessed the group D antigen (Lancefield, 1933). Further, in 1937, Sherman proposed a new taxonomic scheme for the genus Streptococcus and divided them into four groups designated as pyogenic, viridans, lactic and enterococci (Sherman, 1937). It was not until 1984 that the most significant advancement materialized when Shleifer and Klipper-B€alz demonstrated that S. faecalis and S. faecium should be transferred to the newly established Enterococcus genus, which was put forward on the basis of comparative 16S rRNA sequence analysis and DNA–DNA hybridization (Kilpper-Bälz and Schleifer, 1984). And in 1999, Manero and Blanch proposed a biochemical key based on 12 tests that may allow the identification of most of the enterococcal species (Manero and Blanch, 1999). The “faecal streptococci” that were associated with the gastrointestinal tract of humans and animals, with some fermented foods and with a range of other habitats constitute the new genus *Enterococcus*, which is the third-largest genus of LAB after Lactobacillus and Streptococcus.

At present, more than 58 species of the genus Enterococcus have been discovered and are divided into 7 species groups according to the 16S rRNA gene similarity (Graham et al., 2020). However, some reclassifications may occur shortly. For instance, the species *E. solitarius* was recently reclassified as *Tetragenococcus solitarius* (Ennahar and Cai, 2005)*, E. flavescens* was not different enough to be classified separately from *E. casseliflavus*, which was described first and thus had nomenclatural priority (Pompei et al., 1991), and *E. avillorum* was an earlier heterotypic synonym of *E. porcinus* (De Graef et al., 2003)*.* From a medical microbiological, human probiotic and food microbiological point of view, *E. faecium* and *E. faecalis* were still the most important enterococcal species, where these two species were currently the only enterococcal species being utilized. Furthermore, as new species had been identified in 2017, it was likely that the genus Enterococcus had not been fully elucidated and thus may be further reclassified in the future.

## 3 Habitat

The primary habitats of enterococci were the intestines of animals, not only in the gastrointestinal tract of warm-blooded animals (e.g., pigs, dogs, chickens, cows, sheep and donkeys) (Devriese et al., 1991) but also cold-blooded animals (e.g., fish, prawns) (Hirshfeld et al., 2023). Besides, extra-enteric environments such as soil, beach sand, farmland, sewage, surface water, ocean water and vegetation were the secondary habitats due to their tolerance and tough survival ability (Byappanahalli et al., 2012). For instance, enterococci can grow within a broad temperature range of 10–45 °C, survive heat stress up to 60 °C for 30 min, tolerate 6.5 % NaCl, and remain viable in a pH range of 4.5–10. Their adaptability also includes resistance to bile salts (0.3–0.5 %) and desiccation for weeks, supporting their persistence in extra-enteric environments (Fisher and Phillips, 2009). Within the 58 recognized enterococci species, *E. faecalis* and *E. faecium* were the dominant indigenous species in the intestinal tract, with ranges of 10^5^-10^7^ and 10^4^-10^5^ cfu/g feces, respectively (Chenoweth and Schaberg, 1990) (Noble, 1978). Besides, *E. durans*, *E. hirae* and *E. cecorum* also existed in the gastrointestinal tract. High numbers of enterococci in the GI tract of animals and extra-enteric environments affect their existence in foods and probably food quality and safety. Previously, *E. faecalis* and *E. faecium* were indicator microorganisms for poor hygiene and primary contamination with feces, while now the view is proposed that enterococci in food might correlate with indirect contamination by water sources and other environmental factors, and thus should be considered as normal components of animal-derived food microflora (Giraffa, 2003).

## 4 Enterococci in foods

**Fig. 2:**
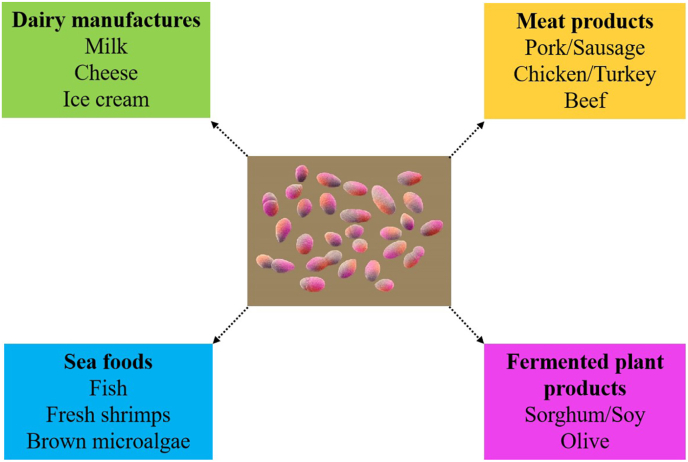
Common sources of enterococci in food systems.

Enterococci are frequently detected in a wide range of food systems, including dairy, meats, fermented plant products, and seafoods (Fig. 2). Beyond their prevalence, their persistence in these niches reflects remarkable ecological adaptations such as tolerance to heat, salt, and acidic environments. In addition, enterococci contribute metabolically through proteolysis, lipolysis, and the generation of flavor-active compounds, thereby influencing ripening and sensory profiles in fermented foods. However, their role in food systems remains controversial because certain strains may provide technological or even probiotic benefits, while others are associated with the dissemination of antimicrobial resistance or the development of opportunistic pathogenicity. The following subsections summarize their occurrence in major food categories, with particular attention to ecological implications, functional contributions, and ongoing debates regarding their safety.

### 4.1 Cheeses

Enterococci were associated with a variety of cheeses, especially manufactured artisanal cheese in Mediterranean countries in southern Europe (Posrtugal, Spain, Italy, and Greece) from raw or pasteurized goat, ewe's, water buffalo, or bovine milk (Franz et al., 1999). Generally, numbers of enterococci in various cheese crudes ranged from 10^4^ to 10^6^ CFU/g and in fully ripened cheeses from 10^5^ to 10^7^ CFU/g (Teuber et al., 1996). In addition, varying counts of enterococci in different cheeses were associated with the cheese type, the extent of milk contamination, the starter, and the technology applied (Litopoulou-Tzanetaki, 2010). The dominance of enterococci in cheeses was attributed to their capacity to survive in hostile environments as well as the production of proteolytic enzymes in connection with casein degradation, which was considered an important factor for cheese ripening (Wessels et al., 1990). What's more, the flavor components such as acetaldehyde and acetoin produced by enterococci also contributed to the aroma development of cheeses (Afzal et al., 2017). The enterococci played a critical role in cheese manufacture and might also have a beneficial effect on the dairy industry to some extent. However, their technological contributions must be weighed against safety concerns, as some cheese-associated strains harbor virulence or resistance determinants, highlighting the dual role of enterococci as both functional ripening agents and potential contaminants.

### 4.2 Meats

High potential for contamination of enterococci in meats at the time of slaughtering was attributed to their presence in the gastrointestinal tract of animals. Enterococci were reported to be the dominant Gram-positive coccal strain isolated from beef and pig cuts as well as chicken samples at mean log counts of 10^4^ to 10^8^ per 100 cm^2^ of carcass surface, among which *E. faecium* and *E. faecalis* were the predominant isolates (Stiles et al., 1978). Besides raw meats, enterococci as thermotolerant bacteria, gained advantage in the heating process of meats accompanied with high core temperature at least 60 °C and even above 70 °C (Ben Braïek and Smaoui, 2019), thus usually associated with salted or cured cooked and processed meats, such as salami in Europe, Landjager in Germany as well as chorizo and espetec in Spain, with counts ranging from 100 to 2.6 × 10^5^ CFU/g (Teuber et al., 1996). However, for the corruption of vacuum-packaged processed meats, higher levels of enterococci were caused by “reworking” in the “secondary-heated” procedure, which was manufactured by adding faulty products (e.g., in which the packaging materials broke during heat treatment) (Franz and Holy, 1996). Their survival under salting, curing, and heat stress illustrates strong metabolic adaptation, but it also raises concerns about the persistence of antibiotic-resistant strains in meat products, making enterococci both resilient ecological members and indicators of processing hygiene.

### 4.3 Fermented plant products

Enterococci were reported present in fermented vegetables due to the fermentation reaction, with the predominance of *E. faecium* and *E. faecalis* in fermented soya, sorghum, and olives (Todorov and Holzapfel, 2015) (Lucena-Padrós et al., 2014). Compared with other enterococci-containing foods, the literature concerning the isolation and characteristics of these products was few and began with some reports in succession as late as 2004. Among these products, table olives were the most one reported, and it was increasingly recognized as a vehicle as well as a source of probiotic bacteria, especially those fermented with traditional procedures based on the activity of indigenous microbial consortia, originating from local environments (Ben et al., 2004) (Randazzo et al., 2004). However, the detailed role of enterococci during fermentation of table olives was scarce to our knowledge, and the only information explicated was that enterococci produced a quicker brine acidification, a greater consumption of carbohydrates and a rapid pH decrease. This metabolic activity suggests that enterococci may contribute to stabilization of plant fermentations, although the limited evidence and lack of safety evaluations keep their functional significance in such systems controversial.

### 4.4 Seafoods

Several enterococcal species, including *E. mundtii*, *E. faecium*, and *E. durans* had been isolated from the viscera and skin of fish (Akter et al., 2020) (Kukułowicz et al., 2024) (El-Jeni et al., 2019). And strains of *E. faecium*, *E. faecalis*, *E. lactis*, *E. casseliflavus*, and *E. gallinarumm* were reported to be isolated from fresh shrimps (Ben BraïEk et al., 2017) (Chajcka-Wierzchowska et al., 2016). Regarding seafoods, the prevalence of enterococci was lower than that in fermented or raw fish. The common isolated strains were *E. faecium*, *E. faecalis*, *E. casseliflavus*, and *E. hirae* (Çardak et al., 2022). Their presence in seafoods may reflect environmental contamination from water sources rather than technological roles, and thus, enterococci in aquatic products are more often considered indicators of hygiene and environmental impact than functional contributors.

## 5 Functional properties of enterococci

### 5.1 Enterocins

Enterocin is a kind of bacteriocin produced by *Enterococcus* spp., usually referring to peptides synthesized from ribosomes with characteristics of cationic, hydrophobic, and heat-stable small molecular weight (containing about 20–60 amino acids) (Gálvez et al., 2007). The most characterized enterocins were produced by *E. faecium* and *E. faecilis* followed by *E. muntii*, *E. avium*, *E. hirae*, and *E. durans*, as well as other species, which could be isolated from various niches, including food, waste, specimens from human infections, feces, or the gastrointestinal tract of humans and animals (Qiao et al., 2020) (Mareková et al., 2003) (İspirli et al., 2015) (Pogány Simonová et al., 2020). Update, based on the main structure, molecular weight, physical chemistry, and molecular properties as well as biological activities, they could be divided into four categories (Franz et al., 2007). Of course, the classification scheme continues to develop with the accumulation of knowledge and emergence of new kinds. Among the four classes, category II accounts for the highest percentage and most of them possessed anti-*Listeria* ability (Oppegård et al., 2007). Enterocins possess broad-spectrum inhibitory activity against putrefactive bacteria and foodborne pathogens (Kasimin et al., 2022), and some have been reported to inhibit Gram-negative bacteria, which represents an unusual property of bacteriocins produced by LAB (Sheoran and Tiwari, 2019). The mechanism of enterocin action is generally similar to that of most bacteriocins, targeting the cell plasma membrane to form pores, thus depleting the transmembrane potential and pH gradient, further leading to the leakage of essential molecules (Krulwich et al., 2011). However, certain enterocins exhibit a distinct mode of action, attacking susceptible bacteria by degrading the cell wall structure, ultimately leading to cell lysis (Nilsen et al., 2003).

Holding the broad-spectrum inhibitory activity against spoilage and food-borne bacteria and being insensitive to chymosin as well as stable in a wide range of pH (2–6) values, enterocin could not only affect rheology and sensory properties, but also extend the shelf-life of fermented food, thus leading them achieving great potential as a biological preservative in the application of food. The first successfully purified enterocin was AS-48 produced by *E. faecalis* A-48-32, which was a circular bacteriocin and was the first enterocin allowed to be applied in the food industry (Grande Burgos et al., 2014), and the application of enterocins in food systems was shown in detail in Table 1. Generally, enterocins could be used as food additives (semi-pururified or purified enterocins as well as antimicrobial films combined with other hurdle technologies) or *in-situ* with live bacteria as fermentation starters and antimicrobial cultures, and the latter means could provide additional benefits, such as the development of flavor and aroma, improvement of maturation process, and the production of bioactive hydrolysates (Khan et al., 2010). However, the assumed pathogenic characteristics of enterococci (the emergence of multiple antibiotic-resistant and virulence genes) made it require a careful safety evaluation before potential biotechnical use, thus making enterococcal bacteriocins produced by heterologous hosts or added as cell-free preparations be considered safer and more practical for application in food preservation (Kawamoto et al., 2002) (García-Vela et al., 2023).

**Table 1:** Utilization of enterocins *in-suit* or *ex-suit* in food systems, including dairy manufactures, meat products, and fruits, as well as vegetable products. “UNK” means “Unknown”.

Food system	Food tested	Producer strains	Enterocin name	Additional treatments	Classification	Molecular mass (Da)	Target organisms	Refs
Dairy manufactures	Munster cheese	*E. faecium* WHE81	Multiple mixture of enterocin (Enterocin A and B)	Co-culture with bacterial supernatant	II	4829, 5479	*L. monocytogenes*	Izquierdo et al. (2009)
	Goat's milk and Jben (Moroccan goat milk's cheese)	*E. faecium* F58	Multiple mixture of enterocin (Enterocin L50)	Adjunct culture	II	5190	*L. monocytogenes*	Achemchem et al. (2006)
	Non-fat hard cheese	*E. faecalis* A-48-32	Enterocin AS-48	Adjunct culture	II	7166	*B. cereus*	Muñoz et al. (2004)
	Half-skimmed milk	*E. faecalis* EJ97	Enterocin EJ97	Co-cultivation; potassium nitrate, sodium nitrite	II	5328	*L. monocytogenes*	García et al. (2004a)
	Skimmed milk and non-fat unripened soft cheese	*E. faecalis* A-48-32	Enterocin AS-48	Adjunct culture; purified enterocin; moderate heat treatment (65 °C, 5 min)	II	7166	*S. aureus*	Muñoz et al. (2007)
	Frankfurters and fresh cottage cheese	*E. casseliflavus* IM 416K1	Enterocin 416K1	Entrapped in an organic-inorganic hybrid coating applied to an LDPE (low-density polyethylene) film	II	<5000	*L. monocytogenes*	Iseppi et al. (2008)
	Ripen cheese	*E. faecalis* L2B21K3 and *E. faecalis* L3A21K6	Multiple mixture of enterocins	Incorporated into gelatin/glycerol films	–	–	*L. monocytogenes*	Silva et al. (2023)
	Fresh Anthotyros whey cheeses	*E. faecium* KE64, *E. faecium* KE82 (MW644969) and *E. faecium* KE118	Enterocin A-B-P extract	Purified enterocin at a concentration of 400 AU/mL	II	4829, 5479, 4493	*L. monocytogenes*	Sameli and Samelis (2022)
	Custard cream	*E. faecalis* N1-33	Enterocin MR-10A	Co-culture with bacterial supernatant	II	5202	*B. cereus*	Hata et al. (2009)
	Soy milk	*E. faecium* CCM 4231	Enterocin CCM 4231	Concentration of 3200 AU/mL	UNK	UNK	*S. aureus* and *L. monocytogenes*	Lauková and Czikková (1999)
	Bryndza (traditional Slovak dairy product)			Concentration of 6400 AU/mL			*L. innocua*, *E. coli* and *S. aureus*	Lauková and Czikková (2001)
	Saint-Paulin cheese			Concentration of 3200 AU/mL			*L.monocytogenes*	Lauková et al. (2001)
	Sunar® (Milk nourishment for suckling babies), Yoghurt			Concentration of 400 AU/mL			*S. aureus*	Lauková et al. (1999a)
	Soft white cheese	*E. faecalis* SRB/ZS/090	Enterocin (E2)	Semi-purified of enterocin at a concentration of 1280 AU/mL; ammonium sulphate	UNK	UNK	*L. monocytogenes*	Vesković-Moračanin et al. (2023)
	Moroccan fresh cheese	*E. hirae* OS1	Enterocin OS1	Combined with essential oils or crude extracts of eight aromatic and medicinal plants at a concentration of 200 AU/g	UNK	UNK	*L. monocytogenes*	Ananou et al. (2023)
Meat products	Spanish-style dry fermented sausages	*E. faecium* CCM 4231 and *E. faecium* RZS C13	Enterocin CCM 4231 and enterocin 13	Starter cultures	II	5477	*Listeria* spp.	Callewaert et al. (2000)
	Raw beef meat	*E. lactis* Q1	Enterocin P	Protective cultures	II	4493	*L. monocytogenes*	Ben Braiek et al. (2020)
	Cacciatore (Italian sausages)	*E. casseliflavus* IM 416K1	Enterocin 416K1	Starter cultures; concentration of 10 AU/g; entrapped in an organic-inorganic hybrid coating applied to an LDPE (low-density polyethylene) film	II	<5000	*L. monocytogenes*	Sabia et al. (2003)
	Dry fermented sausages	*E. faecium* CTC492	Enterocins A and B	Starter cultures; concentration of 256 AU/g	II	4829, 5479	*L. innocua*	Aymerich et al. (2000a)
	Cooked ham			Antimicrobial film at a concentration of 2000 AU/cm^2^; high hydrostatic pressure			*L. monocytogenes*	Marcos et al. (2008)
	Cooked ham blended with distilled water			Concentration of 1280 AU/g; high hydrostatic pressure			*L. monocytogenes*	Garriga et al. (2002)
	Cooked ham, minced pork meat, deboned chicken breasts, pate, slightly fermented sausages			Antimicrobial film at a concentration of 2000 AU/cm^2^; concentration of 648, 1600 and 4800 AU/g			*L. innocua*	Aymerich et al. (2000b)
	Low acid dry fermented sausages			Concentration of 2000 AU/g; High hydrostatic pressure			*L. Monocytogenes* and *S. enterica*	Jofré et al. (2009)
	Dry cured ham and cooked ham			Concentration of 1280 AU/g; high hydrostatic pressure			*L. monocytogenes* and *S. enterica*	Jofré et al. (2008)
	Fish spread	*E. faecalis* BFE 1071	Enterocin 1071 A and B	Crude extract at a concentration of 15000 AU/g	II	4286, 3899	*L. innocua*,	Balla et al. (2000)
							*S. epidermis* and *P. vulgaris*	
	Dry fermented Hornad salami	*E. faecium* CCM 4231	Enterocin CCM 4231	Concentration of 12800 AU/g	UNK	UNK	*L. monocytogenes*	Lauková et al. (1999b)
	Refrigerated raw beef meat	*E. lactis* 4CP3	Enterocin A, B, and P	Protective cultures	II	4829, 5479, 4493	*L. monocytogenes*	Ben Braïek et al. (2018)
	Stored chicken breast meats	*E. lactis* 4CP3, *E. lactis* Q1	Enterocin A, B, and P	Protective cultures			*L. monocytogenes*	Ben Braïek et al. (2019)
	Model meat sasuage	*E. faecalis* A-48-32	Enterocin AS-48	Semi-purified enterocin at concentrations of 112, 225 and 450 AU/g; protective cultures	II	7166	*L. monocytogenes*	Ananou et al. (2005a)
	Model meat sasuage			Semi-purified enterocin at a concentration of 30 or 40 μg/g; protective cultures			*S. aureus*	Ananou et al. (2005b)
	Cooked ham model system			Sodium pyrophosphate combined with 60 μg/g AS-48			*L. monocytogenes*,	Ananou et al. (2010a)
							*S. aureus*	
	Fuet (a low acid fermented sausage)			Concentration of 148 μg/g; high hydrostatic pressure			*L. monocytogenes*,	Ananou et al. (2010b)
							*S. enterica* and *S. aureus*	
	Sardines (Sardina pilchardus)			Under normal atmosphere, vacuum or modified atmosphere			*Staphylococci* spp.	Ananou et al. (2014)
	Ham	*E. durans* 152	Dur 152A and enterocin L50B	Concentration of 400 AU/mL	II	5190, 5178	*L. monocytogenes*	Du et al. (2017)
	Sliced cooked ham	*E. faecium* LM-2	Enterocin LM-2	Concentration of 256 and 2560 AU/g; high hydrostatic pressure	II	3500–6400	*S.enterica* and *L. monocytogenes*	Liu et al. (2012)
	Sliced dry-cured ham	*E. faecium* CTC492	Enterocin A	Polyvinyl alcohol films with Enterocin A	II	4829	*L.monocytogenes*	Aymerich et al. (2022)
Fruit and vegetable products	Alcoholic and non-alcoholic beer	*E. faecium* L50	Enterocins L50A and B	Concentration of 136–3400 BU/mL	II	5190, 5178	*L. brevis*,	Basanta et al. (2008)
							*P. damnosus*	
	Pickled cucumber	*E. faecalis* N1-33	Enterocin MR-10A	Co-culture with bacteria supernatant	II	5202	*L. monocytogenes*	Hata et al. (2009)
	Zucchini puree	*E. faecalis* EJ97	Enterocin EJ97	Concentration of 20 AU/mL; sodium nitrite, sodium benzoate, sodium lactate or sodium tripolyphosphate	II	5328	*B. macroides*,	García et al. (2004b)
							*B. maroccanus*	
	Infant rice-based food	*E. faecalis* S-48	Enterocin AS-48	Concentration range of 20–35 μg/mL	II	7166	*B. cereus*	Grande et al. (2006a)
	Fruit juices			Concentration of 2.5 μg/mL			*A. acidoterrestris*	Grande et al. (2005)
	Apple cider			Concentration of 3 mg/mL			*B. licheniformis*	Grande et al. (2006b)
	Vegetable sauces			25 mg/mL; phenolic compounds (carvacrol, geraniol, eugenol, terpineol, caffeic acid, p-coumaric acid, citral, and hydrocinnamic acid)			*S. aureus*	Grande et al. (2007a)
	Vegetable soups and purees			50 μg/mL; nisin or phenolic compounds (carvacrol, eugenol, geraniol, and hydrocinnamic acid)			*B. ereus*,	Grande et al. (2007b)
							*B. macrolides* and *Paenibacillus* spp.	
	Alfalfa and soybean sprouts, green asparagus			12.5 or 25 mg/mL; chemical preservatives (lactic acid, sodium lactate, sodium nitrite, sodium nitrate, trisodium phosphate, trisodium trimetaphosphate, sodium thiosulphate, n-propyl p-hydroxybenzoate, p-hydroxybenzoic acid methyl ester, hexadecylpyridinium chloride, peracetic acid, or sodium hypochlorite)			*L. monocytogenes*	Molinos et al. (2005)
	Soybean sprouts			25 mg/mL; alkaline pH (9), moderate heat treatment (65 °C for 5 min) and washing with chemical preservatives (EDTA, lactic acid, peracetic acid, polyphosphoric acid, sodium hypochlorite, hexadecylpyridinium chloride, propyl-p-hydroxybenzoate, and hydrocinnamic acid)			*T. enterica*,	Cobo et al. (2008)
							*E. coli* O157:H7, *Shigella* spp.,	
							*E. aerogenes*,	
							*Y. enterocolitica*,	
							*A. hydrophila* and *P. fluorescens*	
	Alfalfa and soybean sprouts, green asparagus			Protective cultures; 50 μg/mL; washing with chemical preservatives (cinnamic and hydrocinnamic acids, carvacrol, polyphosphoric acid, peracetic acid, hexadecylpyridinium chloride and sodium hypochlorite)			*B. ereus*,	Cobo et al. (2008)
							*B. weihenstephenensis*	
	Raw fruits and fruit juices			25 mg/mL; washing with chemical preservatives (trisodium trimetaphosphate, sodium lactate, lactic acid, polyphosphoric acid, p-hydroxybenzoic acid, n-propyl p-hydroxybenzoate, and 2-nitropropanol, Carvacrol and hydrocinnamic acid)			*L. monocytogenes*	Molinos et al. (2008)
	Apple juice			50, 100 and 200 μg/mL; EDTA, sodium tyrophosphate; heat			*E. coli*	Ananou et al. (2005c)
	Apple juice, apple cider			Concentration of 12.5 μg/mL			*L. collinoides*,	Martínez Viedma et al., 2008, Martínez-Viedma et al., 2008
							*L. dioliovorans*,	
							*P. parvulus*	
	Apple juice	*E. faecium* CTC492	Enterocins A and B	30 mg/mL; pulsed-electric field treatment	II	4829, 5479	*S. enterica*	Martínez Viedma et al., 2008, Martínez-Viedma et al., 2008
	Canned fruits and vegetable foods	*E. faecalis* A-48	Enterocin AS-48	Concentration of 6 mg/mL; heat treatment or chemical preservatives (lactic acid, glucose or sucrose)	II	7166	*B. coagulans*	Lucas et al. (2006)
	Zucchini, corn, radishes, mixed salad, carrots, apples, grapes, pineapple, melon	*E. casseliflavus* IM416K1	Enterocin 416K1	Co-culture with bacteria supernatant; chitosan	II	<5000	*L. monocytogenes*	Anacarso et al. (2011)
	Canned corn and canned peas	*E. faecalis* EJ97	Enterocin EJ97	Polythene films coated with the enterococcal bacteriocin enterocin EJ97 (80 AU/mL) alone or in combination with EDTA	II	5328	*B. coagulans*	Martínez Viedma et al. (2010)
	Spoiled banana	*E. faecalis* KT2W2G	Enterocin KT2W2G	Essential oils; cinnamon oil	–	3500–6500	*Lactococcus lactis* subsp.Lactis,	Issouffou et al. (2018)
							*E. Faecalis*,	
							*K. Pneumonia*,	
							*K. Variicola*,	
							*S. marcescens*	
	Oat and soya drinks	*E. faecalis* A-48	Enterocin AS-48	25 μg/mL; phenolic compounds (carvacrol, eugenol, geraniol, and citral) or with 2-nitro-1-propanol (2NPOH)	II	7166	*S. aureus*	Burgos et al. (2015)
	Blueberries			Immersing in a solution of Enterocin AS-48 (50 μg/mL)			*Salmonella*, *E. coli*	Rodríguez López et al. (2023)
	Cherimoya pulp			50 μg/g AS-48 and high hydrostatic pressure			*P. agglomerans*,	Pérez et al. (2015)
							*P. vagans*,	
							*E. gallinarum* and *L. mesenteroides*	

### 5.2 Exopolysaccharides

Exopolysaccharides (EPS) are high molecular weight carbohydrate extracellular biopolymers secreted by various microorganisms, including *Enterococcus* spp. EPS possesses significant functional and biological potentials mainly dependent upon its chemical structure, monosaccharide composition, and chemical groups (Li et al., 2021; Peng et al., 2015; Jurášková et al., 2022). The presence of functional groups (uronic acid, sulfonyl, phosphoryl, succinyl, acetyl, and carboxymethyl), either naturally or chemically modified (sulfonation, phosphorylation, acetylation, and carboxymethylation), contributes to enhancing the biological activity of EPS. The biological activity potential of *Enterococcus* EPS has been widely reported and aroused prodigious interests, including the potential of antioxidant, antibacterial, antibacterial membrane, anti-cancer, immunology, prebiotics and anti-diabetes (the detailed information was listed in Table 2). Those above properties make EPS of enterococci prospective in food, pharmaceutical, biomedical and environmental fields.

**Table 2:** Exopolysaccharides produced by enterococci with different isolation and their functional properties.

Strain	Source	Properties	Refs
*E. faecium* MC13	Gut of fish	Emulsifying, flocculating, antioxidant, and antibiofilm activities	Kanmani et al. (2013)
*E. faecium* MC-5	Gut of fish	Antioxidant and anti-biofilm properties	Tilwani et al. (2021)
*E. faecium* AD1	Puffer fishes	Antioxidant and suppresses cancer cells	Nguyen et al. (2015)
*E. lactis* BE11	Gut of a bee	Antagonism to marine fish pathogens	Zaghloul et al. (2023)
*E. faecalis* DU10	Duck intestine	Antioxidant, anti-biofilm and rheological properties	Venkatesh et al. (2016)
*E. faecium*	Infant's gut	Emulsifying ability, antimicrobial properties	Kumar Bajaj et al. (2015)
*E. faecium* WEFA23	Healthy infant's feces	Antioxidant activity	Jia et al. (2019)
*E. hirae* WEHI01	Healthy infant's feces	Immunomodulatory ability	Jia et al. (2022)
*E. hirae* KX577639	Feces of South Indian Irula tribals	Water solubility index, water holding capacity	Jayamanohar et al. (2018)
*E. faecalis* EJRM152	Human breast milk	Prebiotic property	Kansandee et al. (2019)
*E. faecium* M20	Human blood	Immunomodulatory ability	Arar et al. (2022)
*E. faecalis*	Urinary tract infections	Antibacterial activity and immunomodulatory effect	Abed et al. (2020)
*E. durans* DU1	Iranian dairy product	Anti-biofilm and emulsification activity	Soliemani et al. (2022)
*E. faecium* GRD AA	Milk	Silver nanoparticle synthesis,	Ramasamy et al. (2020)
*E. faecium* AK1247	Traditional fermented milk	Antioxidant activity	Zhao (2021)
	`Kitek		
E. faecium F58	Fresh goat cheese	Anti-adhesion properties against different pathogens	Zanzan et al. (2024)
*E. faecium* (BDU7)	Ngari	Antioxidant potentials	Abdhul et al. (2014)
*E. faecium* K1	Kalarei	Hypocholesterolemic, antioxidant, antibiofilm, and emulsification	Bhat et al. (2020) Bhat and Bajaj (2018)
*E. durans* K48	Kishk	Antioxidant, cell toxicity and antimicrobial activity	Rahnama Vosough et al. (2021)
*E. faecium* R114	Kishk	Prebiotic property	Rahnama Vosough et al. (2022)
*E. faecium* T52	Kishk	Prebiotic property	Rahnama Vosough et al. (2022)
*Enterococcus* sp. F2	Fermented soya beans	Water solubility, water and oil holding capacity, emulsifying activity, rheology	Jiang et al. (2021)
*E. hirae* OL616073	Indian traditional fermented food	Prebiotic property	Kavitake et al. (2024)
*E. faecium* L15	Traditional Korean rice-fermented food containing flatfish	Promoted the differentiation of stem cells	Kim et al. (2022)
*E. hirae* MG6	Sludge and water samples	Antioxidant, cell toxicity, anti-tumor activity and cytokine production	Sharma and Ghosh (2021)
*E. faecalis*	Soil	Antioxidant Activity, and cytotoxicity against HeLa cells	Choudhuri et al. (2020)
*E. faecium* MS79	–	Rheological, antioxidant, antidiabetic, antibacterial properties, antiproliferative	Ayyash et al. (2020)
*E. faecium*	–	Antioxidant capacity, inhibiting pathogens, and suppressing enzymes and cancer cells	Tarique et al. (2024)

### 5.3 Surface layer proteins

The surface (S) layer of bacteria is widely present in Gram-positive and negative bacterial species, as well as *Archaea* (Sára and Sleytr, 2000). It forms the outermost layer of cells, which is a protein cell envelope structure composed of many identical (glycoprotein) subunits, with a molecular weight of 25–200 kDa (Fouet et al., 2010). It forms a two-dimensional, regular, and highly porous array with tilted (p1, p2), square (p4), or hexagonal (p3, p6) symmetry. It was reported that surface layer protein (SLP) had been found in several lactic acid bacteria but not all species, and it was involved in the modulation of stimulation of epithelial barrier function and induction of immune responses as well as change of gut microbiota composition and *etc.* (Bendinelli et al., 2024) (Wang et al., 2018) (Marcos-Fernández et al., 2021). With the dual role of enterococci belonging to LAB, their SLP was generally thought of as a kind of virulence factor in pathogenic species, causing the ability to enhance the adhesion of intestinal cells (Wang et al., 2020). It's precisely because of this, one of our studies appears precious, which demonstrated that SLP from a probiotic *E. faecium* WEFA23 with healthy infant origin could inhibit the infection of *Listeria monocytogenes* CMCC54007 due to it could antagonize the adhesion of pathogenic bacteria to intestinal epithelial cells, thus providing a scientific basis for the application of *E. faecium* WEFA23 as a probiotic candidate strain in microecological additive preparations (He et al., 2021).

### 5.4 Acids producing

Acid production is of significant importance in dairy fermentation, particularly in cheese manufacture, where a rapid decrease of pH (from ∼6.6 to 6.0–5.3 within 6 h at 30 °C) is required for curd formation, flavor development, and suppression of undesirable microorganisms (Graham et al., 2020). Considerable studies on enterococci have shown, however, that these bacteria generally display weak milk-acidifying capacity, as only a small proportion of dairy isolates can reduce pH below 5.0–5.2 even after 16–24 h at 37 °C (Andrighetto et al., 2001; Durlu-Ozkaya et al., 2001; Sarantinopoulos et al., 2001a). The species contribution to acidification remains controversial. *E. faecalis* was once considered the strongest acid producer (Suzzi et al., 2000), but subsequent evidence suggested that *E. faecium* might exhibit higher acidifying activity (Tuncer, 2009). Therefore, further research is needed to completely understand how acid production by enterococci varies by species specificity and to determine which strains are the most acidified. Enterococci mainly produce lactic acid as the dominant metabolite, together with smaller amounts of acetic acid and formic acid (Sarantinopoulos et al., 2001a). These organic acids not only contribute to pH reduction, flavor formation, and microbial safety in dairy products but also play key physiological roles in the intestinal ecosystem by lowering gut pH, suppressing pathogens, and supporting colonization resistance and microbial balance (Caballero-Flores et al., 2023) (Woelfel et al., 2024). Thus, although their milk acidification ability is generally limited and strain-dependent, enterococcal acid production is functionally significant in both dairy fermentation and intestinal homeostasis.

### 5.5 Citrate and pyruvate metabolism

Citrate and pyruvate were present in many raw materials that were used in food fermentation, such as milk, fruits, and vegetables, and were also used as food additives in fermented sausages (Shapiro and Silanikove, 2011). Citrate and pyruvate metabolism were important technological characteristics of many but not all LAB, and their abilities differed from one species to another, producing the volatile and flavor compounds as well as carbon dioxide and contributing to the development of both aroma and texture of dairy products (Hugenholtz, 1993). The fundamental reason for the ability was attributed to the endogenous plasmids, which contained the genes encoding the transporters responsible for the uptake of substrates from the medium.

The data concerning the metabolisms in enterococci remained sporadic and unsystematic compared with the other species in LAB until more studies focused on it in the last few years to the undeniable fact that enterococci, especially *E. faecium* and *E. faecalis*, frequently occur in food such as cheese and milk. Devoyod pinpointed that *E. faecalis* subsp. *Liquefaciens* were able to metabolize citrate when used as the sole carbohydrate as early as 1969 (Devoyod et al., 1968). Gradually, *E. faecalis*, *E. faecium*, and *E. durans* isolated from food were found to exhibit strain-to-strain abilities to utilize citrate or pyruvate as the sole carbon sources, with the former one achieving the best ability in the organic acids (Rea and Cogan, 2003) (Sarantinopoulos et al., 2003) (Cabral et al., 2007). Classically, Sarantinopoulos focused on the citrate metabolism of *E. faecalis* FAIR-E 229 in various growth media containing citrate either in the presence of glucose or lactose or as the sole carbon source, and the results showed that citrate and lactose co-metabolized in milk while the citrate was not catabolized in the presence of either lactose or glucose in MRS broth although the initial content of citrate was added. Of course, when present as the sole carbon source, citrate was catabolized and the main end products were acetate and formate (Sarantinopoulos et al., 2001b). Moreover, the kind of volatile compounds (diacetyl, acetoin and 2,3 butanediol) produced by the breakdown of lactose and citrate during cheese ripening might further contribute to flavor. The aforementioned studies indicated that enterococci possess the metabolic potential, which positively contributed to the distinctive organoleptic properties of fermented dairy products to some degree.

### 5.6 Proteolysis and lipolysis

#### 5.6.1 Proteolysis

Protein degradation is related to the proteolytic and peptide-hydrolytic activities of microorganisms. Casein degraded through proteolytic activity plays an important role in the development of texture and flavor of cheese ripening and fermented milk products. Alike, some peptides produced after peptide hydrolytic also contribute to the formation of flavor, while other unpopular bitter peptides cause odor. Moreover, the secondary degradation of amino acids was also believed to have a significant impact on the flavor development of cheese (Foulquié Moreno et al., 2006). Compared with *Lactococcus* and *Lactobacillus*, which were more studied in LAB species, the data about the proteolysis system of enterococci remained sporadic, and this biochemical characteristic might be the most contradictory characteristic of this genus. The research about proteolytic and peptide hydrolytic activities of enterococci began in 1969 (Somkuti and Babel, 1969), among which some authors reported a high level of proteolytic activity of it (Durlu-Ozkaya et al., 2001) (Tulini et al., 2016), while others believe that it usually showed a weak proteolytic activity (Morandi et al., 2006) (Sarantinopoulos et al., 2002). The consensus between them lay that they all believe the majority of proteolytic strains belong to *E. faecalis*, especially from food sources, though the ability remained strain and condition specific, which was consistent with the observation that *gelE* gene, which led to casein hydrolysis during the growth of enterococci in milk was more common in *E. faecalis* than in other species (Zhang et al., 2016). It was further found that calcium lactate and some inorganic phosphates [except that lower NaCl concentration (2 %, w/v) had a positive effect on casein decomposition, while higher NaCl concentration hurt casein decomposition] did not affect casein hydrolysis, which reached the maximum at the conditions of isoelectric point 4.6, pH 7.5 and 45 °C (Hegazi, 1990). In addition to the natural milk protein hydrolase and renin used for protein degradation, the cell wall-related protease of LAB and the cell endopeptidase released after cell lysis in curd were considered to play an important role in the hydrolysis of casein during cheese preparation (Wilkinson et al., 1994). Moreover, hydrolases of enterococci have also been demonstrated with the ability to produce bioactive milk hydrolysates, especially in the form of bioactive peptides (Graham et al., 2020). Therefore, it is of great importance to evaluate the proteolytic activity of enterococci isolates for their further application in the food industry.

#### 5.6.2 Lipolysis and esterase activities

Esterases are classified as enzymes that hydrolyze substrates in solution, while lipases hydrolyze substrates in lotion (Giraffa, 2003). Though the capacity of lipase and esterase in enterococci was generally considered as weak compared with other microbial groups, the contribution to the development of flavor and texture of cheese was an accepted fact even if the concrete role of lipase and esterase to cheese ripening was not clear. Alike to the characteristics aforementioned, data on the lipolytic activity of enterococci remained limited and often conflicting. Contradictory data on the lipolytic activity of enterococci were reflected in the research results of Durlu Ozkaya et al. who concluded that this genus possessed obvious lipolytic activity (Durlu-Ozkaya et al., 2001), while Villani and Coppola demonstrated low lipolytic activity of it (Villani and Coppola, 1994). For species with the strongest hydrolysis ability, the studies also seemed to be inconclusive. Sarantinopoulos et al. reported that *E. faecium* had the highest lipolytic activity, followed by *E.durans*, while *E. faecalis* had the weakest lipolytic activity (Sarantinopoulos et al., 2001b). However, in the observation of esterase hydrolysis activity, the esterase activity of *E. faecalis* was the highest, while there was no significant difference between *E. faecalis* and *E. faecium*. Moreover, the ester solubility of enterococci was considered to be higher than that of most lactic acid bacteria (Tsakalidou et al., 1994). Therefore, the lipolytic enzyme and esterase activity of enterococci seemed to be strain and/or region-specific, and their ester decomposition system was more complex and efficient than the lipid decomposition system.

## 6 Enterococci as probiotics

### 6.1 Starter/adjuncts cultures

Enterococci were used as starter cultures or starter adjuncts in the food industry, especially in milk manufacturers, with the advantage of accelerating ripening and producing ideal flavors. For instance, the Advisory Committee on Novel Foods and Processes allowed the use of *E. faecium* strain K77D as a starter culture in fermented dairy products (Hanchi et al., 2018). Moreover, some strains of enterococci with specific antimicrobial activity against pathogenic or spoilage bacteria could be used as protective cultures. Several studies demonstrated the inhibitory effect of enterocin-producing *E. faecium* or *E. faecalis* against *L. monocytogenes* and *S. aureus* artificially contaminating food systems (Farias et al., 2021) (Rocha et al., 2019) (Sonbol et al., 2020). Apart from being used as starter cultures in dairy food, enterococci could also be applied to ferment some food and drug homology to enhance the inherent biological activity. *E. faecim* WEHI01, isolated from healthy newborn infants, was used to ferment lotus (*Nelumbo nucifera* Gaertn.) leaf and thus presented its stronger anti-adipogenic effect than unfermented lotus leaf when evaluated in 3T3-L1 preadipocytes and high-fat diet (HFD)-induced obese rats, which might be related to the enhanced antioxidant capacity and regulatory effect of intestinal flora of rats (He et al., 2022). Similarly, Xie et al. demonstrated that Xuefeng black-bone chickens fed with a basal diet fermented by a mixed cocktail of *E. faecium*, *Bacillus subtilis*, *Saccharomyces cerevisiae*, *Lactiplantibacillus plantarum*, and gained improved intestinal morphology, including increased villus height and its ratio to crypt depth, and decreased crypt depth of the jejunum (Xie et al., 2020).

### 6.2 Inhibiting the development of cancer

Cancer, generally refers to malignant tumors, was closely related to environmental factors (smoking, alcoholism, obesity, sedentary lifestyle, diabetes mellitus, consumption of red meat, a high-fat diet and inadequate intake of fiber) (Lewandowska et al., 2019). It was reported by WHO that there were 19,776,499 new cases and 9,743,832 new deaths of malignant tumors worldwide in 2022 (Global cancer burden growing, 2024).

Classical therapy for cancer includes surgery, chemotherapy and radiation. In the last decades, as a promising supplement therapy, LAB including enterococci demonstrated protective effects against cancer mainly through modulating the mucosal immune system and changing the expression of some host genes associated with important functions (nutrient uptake, metabolism, angiogenesis and mucosal barrier protection) (Grenda et al., 2022). To date, there have been considerable reports on the role of enterococci in cancer treatment, mainly focused on colorectal cancer (CRC) and melanoma, *etc*. An *in vitro* study demonstrated that *E. faecilis* LMG 7937, when co-cultivated with HCT-116 (an aggressive CRC lineage), was capable of down-regulating the mRNA expression of angiopoietin-like protein 4, which was normally associated with the development of some cancers (Grootaert et al., 2011). Moreover, when stimulated with silver nanoparticles (AgNPs), led to an increase in the extracellular folate concentration, *E. durans* enhanced the capacity to increase cancer cell death and decreasing the viability of cancer cells (Bhalla et al., 2022). Certainly, there are also some clues reflecting the anti-CRC of enterococci *in vivo*. For example, Miyamoto et al. demonstrated that administration of heat-killed *E. faecalis* EC-12 reduced polyp development in the proximal to middle portion of the small intestine in Min mice (APC-mutant mice) by suppressing β-catenin signaling (Miyamoto et al., 2017). Apart from CRC, Yang et al. demonstrated infant-derived *E. faecium* WEFA23 was capable of inhibiting the development of melanoma *via* the inhibition of proliferation and inflammation as well as the promotion of apoptosis (Yang et al., 2023). In addition, it had been found that the efficacy of the anti-cancer immunomodulatory agent relied on intestinal bacteria. Griffin et al. demonstrated that *Enterococcus* supported immunotherapy of PD-L1 in melanoma by secreting orthologs of NlpC/p60 peptidoglycan hydrolase SagA that generated immune-active muropeptides (Griffin et al., 2021). Another typical strain was *E. hirae*, which translocated from the small intestine to secondary lymphoid organs and increased the intratumoral CD8/Treg ratio, triggered specific-memory Th1 cell immune responses, activated the autophagy machinery in enterocytes and mediated ATG4B-dependent anticancer effects, and shifted the host microbiome toward a *Bifidobacteria*-enriched ecosystem, thus selectively extended progression-free survival in advanced lung, spleen, melanoma and ovarian cancer patients treated with chemo-immunotherapy (CTX and PD-L1) (Daillère et al., 2016). Moreover, the concrete substances of enterococci functioned in anti-cancer progress also gained much more attention, enterocin was the most studied one e.g., Enterocin 12a, LNS 18, NKR-5-3B. The researchers proposed that the enterocin was a potential destroying agent and achieved acceptable apoptotic effects on the HEPG-2, AGS, HeLa, MCF-7, and HT-29 human cancer cell lines with negligible side effects on the assayed non-cancerous (HUVEC) cell line (Sharma et al., 2021) (Al-Madboly et al., 2020). In addition, the effect of other substances e.g., acetic acid, acetoin, and diacetyl, and less lactic acid also attracted the interest of researchers (Jiao et al., 2022).

### 6.3 Inhibiting the growth of food-borne pathogens

Enterococci were proven to inhibit 9 common food-borne pathogens e.g., *L. monocytogenes*, *S. aureus* and *Salmonella* et al., thereof *L. monocytogenes* was well documented. *L. monocytogenes* can grow in diverse niches of environmental conditions and tolerate hostile conditions, making it difficult to be eradicated from food industrial facilities and persist on equipment, utensils, floors, and drains, thus ultimately reaching food products by cross-contamination. Once consumed with the contaminated foods, outbreaks of disease with high mortality induced by *L. monocytogenes* will probably occur (Farber and Peterkin, 1991).

One strategy is the application of bacteriocins or bacteriocin-producing cultures for food bio-preservation. Achemchem et al. showed that *E. faecium* F58 originated from goat's milk and reduced *L. monocytogenes* by 1–4 log units when cultivated with enterocin-producing strains at the start of cheese preparation on Jben (Moroccan fresh cheese), and it completely inhibited *L. monocytogenes* when left to grow for 12 h before contamination with *L. monocytogenes* in whole milk (Achemchem et al., 2005). Similarly, enterocin-producing strain *E. faecium* 7C5 and *E. faecium* WHE81 were also capable of inhibiting the growth of *L. monocytogenes* on the surface of Taleggio (Italian soft smear cheese) and rind of Munster cheese (a red-ripened soft cheese), respectively, and the latter reduced the count of *L. monocytogenes* to an undetected level on the 7 days of ripening (Izquierdo et al., 2009). Besides *E. faecium*, Cavicchioli et al. demonstrated that *E. hirae* ST57ACC controlled the growth of the *L. monocytogenes* 422 in the matrix after 48 h when cultivated with the pathogen in skim milk (Cavicchioli et al., 2017). In addition to milk products, meat products were another kind always contaminated with *L. monocytogenes*. Callewaert et al. showed that *E. faecium* CCM 4231 and *E. faecium* RZS C13 of non-meat origin produced a bacteriocin inactive against other LAB but active against *Listeria* spp. when used as starter cultures in sausage fermentation (Foulquié Moreno et al., 2003). Similarly, cultivation with *E. casseliflflavus* IM 416K1 Bac ^+^ inhibited *L. monocytogenes* NCTC 10888 in Italian sausages (Cacciatore) to an undetectable level and the inhibitory effect was related to the anti-listerial enterocin termed enterocin 416K1 (Sabia et al., 2003). Interestingly, besides considerable studies about bacteriocins, Chen et al. revealed that the surface layer protein (SlpB) isolated from *L. crispatus* enhanced the inhibitory activity of nisin against *Staphylococcus saprophyticus*, extending the shelf-life of chicken meat (Sun et al., 2017). Alike, He et al. showed that the surface layer protein of *E. faecium* WEFA23 inhibited the adhesion and invasion of *L. monocytogenes* both in epithelial cell Caco-2 and C57BL/6J mice (He et al., 2021), which provided a scientific basis for the application of *E. faecium* WEFA23 as a probiotic candidate strain in microecological additive preparations.

### 6.4 Ameliorating the syndrome of metabolic syndrome

Metabolic syndrome (MetS) is a complex collection of metabolic disorders, including obesity, hyperglycemia, hypertension, hypercholesterolemia, high blood viscosity, hyperuricemia, high-fat liver, and hyperinsulinemia, which is commonly found in middle-aged and elderly populations, and increases gradually in youth (Huang, 2009). With the development of omics technology, MetS was confirmed to be closely related to an unbalance of intestinal flora. Accumulative data in the recent decades showed that probiotics (i.e., *Enterococcus*, *Lactobacillus*, and *Bifidobacterium*) originated from healthy individuals have been proven to improve chronic diseases and metabolic syndromes (Jiang et al., 2023).

Type 2 diabetes mellitus (T2DM) results from insulin resistance or relative insulin deficiency, leading to syndromes of retinopathy, nephropathy, neuropathy, and cardiovascular complications with protracted hyperglycemia (Zheng et al., 2018). Studies about the anti-diabetes effect of LAB *in vivo* and *in vitro* were mainly focused on *L. plantarum*, *L.casei*, *L. paracasei* and *L. reuteri etc* (Zhong et al., 2024; Wang et al., 2017; Ma et al., 2025; Gu et al., 2023). Distinctively, Wei et al. proposed that *E. hirae* WEHI01 from a healthy infant improved obesity and T2DM in rats, mainly through improving the serum lipids, regulating glycolipid metabolism in the liver and modifying the gut microbiota (Wei et al., 2020). Alike, intervention of *E. faecium* CI1 with inulin alleviated the syndrome of type II diabetes in high-fat diet-induced *Drosophila melanogaster* model (weight, larva size, crawling speed and climbing, as well as lipid deposition and micronuclei number in the gut) by down-regulating insulin-like genes (*Dilp 2*, *Dilp 5*) and the reactive oxygen species level (Bhanja et al., 2022). Moreover, a systematic review and meta-analysis of randomized controlled clinical trials conducted by Bock et al. demonstrated supplementation with probiotics, prebiotics or symbiotics improved metabolic variables in individuals with diabetes mellitus, suggesting its potential role as an adjuvant treatment in improving metabolic outcomes (Bock et al., 2021).

**Fig. 3:**
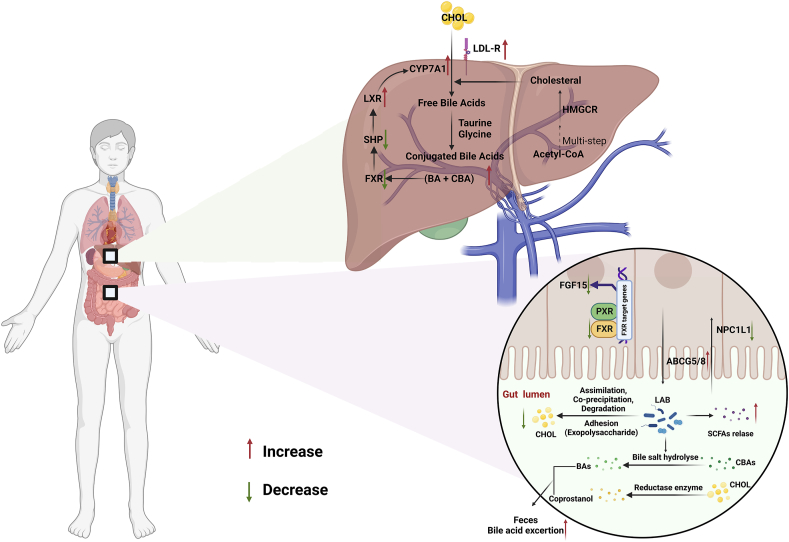
Schematic diagram of the cholesterol-lowering mechanisms of enterococci. The potential mechanisms underlying the reduction of the cholesterol effect of enterococci could be summarized into the following points. (1) Enterococci could decrease cholesterol *via* the assimilation of cholesterol, coprecipitation of cholesterol with deconjugated bile, the binding of cholesterol to probiotic cell walls, and the incorporation of cholesterol into probiotic cell membranes during growth. (2) Some specific enzymes such as bile salt hydrolase (BSH) and reductase, which were beneficial to the transition of conjugated bile acids (CBA) to bile acids (BA), and cholesterol to coprostanol, respectively. (3) Some metabolites of enterococci such as exopolysaccharides could modulate the gastrointestinal microbiome and produce short-chain fatty acids (SCFAs) that inhibit the activity of HMGCR, thus decreas the synthesis of cholesterol. (4) Enterococci could modulate key cholesterol transport by down-regulating expression of NPC1L1, ABCA1, CD36, and SR-B1 *via* modulating expression of critical receptors such as FXR, LXR, SHP and LDL-R. These mechanisms were speculated based on existing experimental results and further studies are needed to elucidate which ones play dominant roles. **Abbreviation:** LAB: lactic acid bacteria; CHOL: cholesterol; BA: bile acids; CBA: conjugated bile acids; CYP7A1: cholesterol 7 alpha-hydroxylase; LDL-R: low-density lipoprotein receptor; LXR: liver X receptor; SHP: small heterodimer partner; FXR: farnesoid X receptor; HMGCR: 3-hydroxy-3-methylglutaryl-coenzyme A reductase; FGF15: fibroblast growth factor 15; NPC1L1: niemann-pick C1-like 1; ABCG5/8: ATP-binding cassette subfamily G member 5/8.

Hypercholesterolemia, a pathological condition characterized by an exaggerated rise in serum cholesterol, is one of the main risk factors in the development of atherosclerosis and cardiovascular diseases (CVDs) (Civeira et al., 2022). The common treatment for ameliorating hypercholesterolemia includes drugs of statins, ezetimibe, proprotein convertase subtilisin/kexin type 9 (PCSK9), and peroxisome proliferatively activated receptor (PPARα) inhibitors, achieving a good effect; however, their side effects (e.g., diarrhea, memory dysfunction, and diabetes risk) should not be neglected (Ruscica et al., 2023). Update, LAB, especially bile salt hydrolase (BSH)-active species, has been demonstrated capable of cholesterol-lowering efficacy. For example, *E. faecium* GEFA01 from healthy lean individuals and *E. faecium* WEFA23 from new-born infants were proved to decrease cholesterol levels, mechanisms of the progress included: 1) regulating the expression of genes relevant to the decomposition (CYP7A1), synthesis (HMGCoAS, SCD1), and transportation (LDLR, SCARB1) of cholesterol, 2) remodeling the intestinal microbiota similar to that in rats fed with normal diet, 3) modulating the gut microbiota-SCFA axis (Xu et al., 2022) (Zhao et al., 2023). Alike, several reports were indicating that treatment with enterococci (including *E. faecium*, *E. faecilis* and *E. durans*) alone or a bacterial cocktail mixed with other probiotics such as *Limosilactobacillus reuteri, Bififidobacterium animalis* and *Lactiplantibacillus plantarum* revealed an effect in improving hypercholesterolemia (Table 3) and the potential mechanisms underlying the activity were systemically summarized in Fig. 3. With the aid of bio-information, Aswal et al. compared a rhizospheric *E. faecium* LR13 cholesterol-assimilating probiotic with *E. faecium* WEFA23 by comprehensive genomic analysis and the results showed that 21 unique proteins absent in non-cholesterol assimilating probiotics existed in both strains and 14 proteins of these directly helped in cholesterol assimilation by producing short-chain fatty acids, lipid (sterol) transport and membrane stabilization, and bile salt hydrolase activity (Aswal et al., 2023). Besides enterococci, products fermented (e.g., fermented soy extract and milk) with the genus were also reported with the capacity to lower cholesterol, which is also in Table 3 in detail.

**Table 3:** Treatment of enterococci in hypercholesterolemia and the potential mechanism.

Strain (Source)	Intervention	Properties	Mechanisms	Refs
*Enterococcus faecium* GEFA01 (Healthy lean man)	C57BL/6J	The body weight of mice and the levels of serum TC, LDL-C, hepatic TC, TG, and LDL-C decreased, and the serum HDL-C increased	Downregulated the gene expression of *Hmgcr*, *Srebp-1c*, *Fxr*, *Shp*, and *Fgf15*, upregulated the gene expression of *Ldlr*, *Abcg5/8*, *Abca1*, *Cyp7a1*, and *Lxr* in the liver; increased the relative abundance of *Lactobacillus*, *Akkermansia*, *Bifidobacterium*, and *Roseburia*, and decreased the abundance of *Helicobacter*	Xu et al. (2022)
	1 × 10^9^ CFU			
	8 weeks			
*Enterococcus faecium* strain 132 (Human fecal samples)	Sprague-Dawley rats	The serum TC, TG, LDL-C, liver TBA, ALT, AST decreased	Regulated the expression of the *CYP8B1*, *CYP7A1*, *SREBP-1*, *SCD1* and *LDL-R* gene; decreased the abundance of *Veillonellaceae*, *Erysipelotrichaceae* and *Prevotella*; increased fecal acetic acid and propionic acid	Yang et al. (2021)
	1 × 10^9^ CFU			
	8 weeks			
*Enterococcus durans* HS03 *Enterococcus lactis* YY1 (Soft chhurpi)	Sprague-Dawley rats	The serum TC, TG, LDL-C and VLDL decreased, and the serum HDL-C increased	The liver MDA, GSH, and CAT activities decreased; the fecal *lactobacilli* increased and *E. coli* decreased	Ghatani et al. (2022)
	1 × 10^8^ CFU			
	3 weeks			
*Enterococcus faecalis* ATCC19433	C57BL/6J	The serum and liver TC decreased	Increased the expression of *ABCG5/8*; increased the abundance of *Lactobacillus*, *Bifidobacterium*, and *Akkermansia*	Zhu et al. (2019)
	1 × 10^9^ CFU			
	4 weeks			
*Enterococcus faecium* WEFA23 (Feces of newborn infants)	C57BL/6J	The serum TC and LDL-C decreased	Upregulated cholesterol metabolism (*Cyp27a1*, *Cyp7b1*, *Cyp7a1*, and *Cyp8b1*) levels in the liver, cholesterol transportation (*Abca1*/5/8) in the ileum and liver, and downregulated *Npc1l1*; the relative abundance of *Allobaculum*, *Blautia*, and *Lactobacillus* increased; and the ratio of *Firmicutes* to *Bacteroidetes* (F/B), *Lachnoclostridium* and *Desulfovibrio* decreased	Zhao et al. (2023)
	1 × 10^9^ CFU			
	12 weeks			
*Enterococcus faecium* TF-18 (Traditional Thai foods)	Wistar rats	The serum TC, TG, and LDL-C decreased,the AST/ALT ratio increased	The abundance of *Lactobacillus*, *Bifidobacterium*, *Enterococcus*, *Akkermansia*, and *Ruminococcaceae* increased	Puttarat et al. (2023)
	1 × 10^9^ CFU			
	8 weeks			
Enterococcus faecium R-026 Bacillus subtilis R-179	C57BL/6	The serum TC, LDL-C decreased	The relative abundance of *Actinobacteriota*, *Colidextribacter*, and *Dubosiella* decreased	Huang et al. (2023)
	0.023/0.230 g/mouse/day (Each gram of powder contained 4.4 × 10^9^ CFU of *B. subtilis* R-179 and 6.0 × 10^10^ CFU of *E. faecium* R-026)			
	5 weeks			
*Enterococcus faecium* Cb5 (Traditional corn beer)	Wistar albino rats	The serum LDL-C decreased and HDL-C increased	–	Fossi et al. (2022)
	1 × 10^8^ CFU			
	4 weeks			
*E. durans* KLDS 6.0930 (Traditional naturally fermented cream in China)	Sprague-Dawley rats	The serum TC and LDL-C decreased, and fecal TBA increased	–	Guo et al. (2016)
	2 × 10^9^ CFU			
	4 weeks			
*Enterococcus faecium* CRL 183 *Lactobacillus helveticus* 416 (Bean plant extract)	New Zealand white rabbits	The serum TC, non-HDL-C and ox-LDL Ab decreased, and the serum HDL-C increased	The abundance of *Lactobacillus* spp., *Bifidobacterium* spp. and *Enterococcus* spp. Increased and that of *Enterobacteria* decreased	Cavallini et al. (2011)
	The aqueous soy extract fermented with *E. faecium* CRL 183 and *L. helveticus* 416			
	60 days			
*Enterococcus faecium* CRL 183 *Lactobacillus helveticus* 416 (Bean plant extract)	Wistar rats	The circulating lipid levels decreased	–	Cheik et al. (2008)
	The aqueous soy extract fermented with *E. faecium* CRL 183 and *L. helveticus* 416			
	8 weeks			
*Enterococcus faecium* CRL 183 *and Lactobacillus helveticus* 416 (Bean plant extract)	New Zealand rabbits	The serum TC decreased and the serum HDL-C increased	–	Rossi et al. (2000)
	The aqueous soy extract fermented with *E. faecium* CRL 183 and *L. helveticus* 416			
	30 days			
*Enterococcus faecium* CRL 183 *and Lactobacillus helveticus* 416 (Bean plant extract)	Moderately hypercholesterolemic men	The serum TC, non-HDL-C, LDL-C decreased, the serum HDL-C increased	The increasing equol production	Cavallini et al. (2016)
	Isoflavone-supplemented soy product fermented with *E. faecium* CRL 183 and *L. helveticus* 416			
	42 days			
*Enterococcus faecium* (EF) M-74 (Soil)	Moderately hypercholesterolemic men	The serum TC and LDL-C decreased	–	Hlivak et al. (2005)
	2 × 10^9^ CFU *E. faecium* M-74 plus 50 microg of organically bound selenium			
	60 weeks			
*Enterococcus faecium* CRL183 (Bean plant extract)	New Zealand white rabbits	The serum TC decreased and the serum HDL-C increased	–	Cavallini et al. (2009a)
	10^8^ CFU			
	60 days			
*Enterococcus faecium* CRL183 (Bean plant extract)	New Zealand White rabbits	The serum ox-LDL Ab decreased, and the serum HDL-C increased	–	Cavallini et al. (2009b)
	Isoflavone-supplemented soy yogurt fermented with *E. faecium* CRL183			
	60 days			
*Streptococcus thermophilus* *Enterococcus faecalis* (Intestinal flora of inhabitants of Abkhasia)	Non-obese, normocholesterolaemic	The serum TC and LDL-C decreased	–	Agerbaek et al. (1995)
	Danish men, aged 44 y old			
	Fermented milk product contained *E. faecium* and two strains of *S. termophilus*			
	6 weeks			
*Streptococcus thermophilus* *Enterococcus faecalis* (Intestinal flora of inhabitants of Abkhasia)	Non-obese and normocholesterolemic females and males, aged 50–70 y old	The serum TC and LDL-C decreased	–	Richelsen et al. (1996)
	Fermented milk product contained *E. faecium* and two strains of *S. termophilus*			
	6 months			
*Streptococcus thermophilus* *Enterococcus faecalis* (Causido)	Mild to moderate primary hypercholesterolemia	The serum TC and LDL-C decreased	–	Bertolami et al. (1999)
	Product of fermented milk (Gaio)			
	8 weeks

## 7 Conclusion and future perspectives

Enterococci are ubiquitous lactic acid bacteria present in the gastrointestinal tract of humans and animals, diverse environments, and traditional fermented foods. They play important roles in food quality by enhancing flavor and organoleptic properties, producing bacteriocins against pathogens such as *Listeria monocytogenes*, and inhibiting spoilage organisms, while also exhibiting probiotic potential with health-promoting effects, including modulation of gut microbiota, immune responses, and mitigation of metabolic syndrome. Despite substantial progress in understanding their metabolic traits, antimicrobial activities, and applications in fermented foods, critical knowledge gaps remain, particularly regarding their safety in food and probiotic applications, the effects of processing and fermentation on their viability and bioactive compound production, and the molecular mechanisms underlying intestinal adhesion, immune cross-talk, and gut microbiota modulation. Future research should systematically address these gaps by employing omics and systems biology approaches to link genomic traits with functional probiotic properties while ensuring the absence of virulence factors. Moreover, integrating artificial intelligence and machine learning can facilitate high-throughput screening and prediction of safe, functional enterococcal strains, supporting the development of next-generation probiotics and expanding their applications as functional ingredients in food systems, ultimately advancing both food innovation and human health promotion.

## CRediT authorship contribution statement

Yao He: Investigation, Visualization, Writing – original draft, Writing – review & editing; Zhigao Liu: Investigation, Validation, Visualization; Yina Huang: Formal analysis, Visualization; Liang Qiu: Investigation, Supervision, Validation; Xueying Tao: Conceptualization, Supervision, Validation, Writing – review & editing; Hua Wei: Conceptualization, Funding acquisition, Project administration, Supervision, Validation, Writing – review & editing.

## Declaration of competing interest

The authors declare that they have no known competing financial interests or personal relationships that could have appeared to influence the work reported in this paper.

## Data Availability

No data was used for the research described in the article.
